# CAR T-Cells for the Treatment of B-Cell Acute Lymphoblastic Leukemia

**DOI:** 10.3390/jcm12216883

**Published:** 2023-10-31

**Authors:** Khalil Saleh, Florence Pasquier, Camille Bigenwald, Stéphane De Botton, Vincent Ribrag, Cristina Castilla-Llorente

**Affiliations:** 1 International Department, Gustave Roussy Cancer Campus, 94800 Villejuif, France; khalil.saleh@gustaveroussy.fr; 2 Department of Hematology, Gustave Roussy Cancer Campus, 94800 Villejuif, France; florence.pasquier@gustaveroussy.fr (F.P.); camille.bigenwald@gustaveroussy.fr (C.B.); stephane.debotton@gustaveroussy.fr (S.D.B.); vincent.ribrag@gustaveroussy.fr (V.R.); 3 Département D’innovation Thérapeutique et D’essais Précoces (DITEP), Gustave Roussy Cancer Campus, 94800 Villejuif, France

**Keywords:** B-cell acute lymphoblastic leukemia, blinatumomab, tisagenlecleucel, brexucabtagene autoleucel, cytokine release syndrome, immune effector cell-associated neurotoxicity syndrome

## Abstract

B-cell acute lymphoblastic leukemia (B-ALL) is the most common subtype of acute leukemia in the pediatric population. The prognosis and treatment of B-ALL have dramatically improved over the past decade with the adoption of intensive and prolonged combination chemotherapy regimens. The advent of novel immunologic agents such as blinatumomab and inotuzumab has changed the treatment landscape of B-ALL. However, patients have continued to relapse, raising the need for novel therapies. Chimeric antigen receptor (CAR) T-cells have achieved a milestone in the treatment of B-ALL. Two CD19-targeting CAR T-cells were approved by the Food and Drug Administration and the European Medicines Agency for the treatment of relapsed and/or refractory B-ALL. In this review, we review the available data regarding CD19-targeting CAR T-cells with their safety profile as well as the mechanism of resistance to these agents and the way to overcome this resistance.

## 1. Introduction

B-cell acute lymphoblastic leukemia (B-ALL) is the most frequent subtype of acute leukemia in children with an annual incidence in the United States of nearly 3000 patients per year [1]. In Europe, the estimated overall incidence is 1.28 per 100,000 individuals annually with significant age-related variations [2]. The treatment of B-ALL has changed drastically over the past decade, and the overall survival (OS) has dramatically improved. The adoption of intensive and prolonged combination chemotherapy regimens has led to the majority of pediatric patients with ALL being cured, with an overall survival (OS) exceeding 85% [3,4,5,6,7]. In young adult populations, pediatric-inspired regimens are associated with improved relapse-free survival (RFS) and OS in comparison with historical data, with an OS ranging up to 50–60% [8,9,10]. Frontline induction chemotherapy in B-ALL is associated with high rates of complete remission (CR), nearly 90%, but approximately 40 to 50% of adult patients will relapse. The treatment of relapsed/refractory (R/R) B-ALL remains challenging, and patients have a dismal prognosis with conventional chemotherapy with CR rates of 30 to 40% after first-line salvage treatment and 10% with second-line salvage therapy [11,12,13,14]. The advent of novel immunotherapies such as blinatumomab (CD19-targeting bispecific T-cell engager) and inotuzumab ozogamicin (anti-CD22 antibody–drug conjugate) is associated with significantly prolonged event-free survival (EFS) and OS in both upfront and R/R disease continuing to change the treatment paradigm of patients with B-ALL [15,16,17]. However, the durability of response in these two clinical trials was limited, patients died within 24 months, and only patients who proceeded to allogenic hematopoietic stem cell transplantation (HSCT) achieved long-term survival [18]. More recently, the development of anti-CD19 chimeric antigen receptor (CAR) T-cell therapy achieved a major milestone in the treatment of R/R B-ALL and added another option to the arsenal against B-ALL. Two CD19-targeting CAR T-cells are currently approved by the Food and Drug Administration (FDA) and the European Medicines Agency (EMA) for the treatment of R/R B-ALL: tisagenlecleucel (CTL019) in children and young adults up to 25 years of age and brexucabtagene autoleucel (KTE-X19) in adult patients (aged more than 26 years for EMA), based on the results of the ELIANA and ZUMA-3 trials, respectively [19,20]. The median OS in the majority of trials evaluating CD19-targeting CAR T-cells is beyond 12 months. However, nearly 50% of patients presented disease relapse within 1 year [19,21]. This relapse may be due to loss of CAR T-cell persistence or the loss of the CD19 antigen due to epigenetic alterations or acquired genetic mutations [22,23]. In this paper, we review the major trials evaluating CD19-targeting CAR T-cells, the mechanisms of resistance to these agents, and the utility of alternative solutions such as bispecific CAR T-cells. We also discuss the role of consolidative allogenic HSCT and the toxicity and management of CAR T-cells in B-ALL.

## 2. CD19-Targeting CAR T-Cells

The major clinical trials evaluating anti-CD19 CAR T-cells in patients with R/R B-ALL are summarized in Table 1. One of the first CD19-targeting CAR T-cells was developed by the National Cancer Institute (NCI) and was used for R/R B-ALL in children and young adults. In the initial report, 21 patients were enrolled, and 19 patients received the prescribed dose. This phase 1 dose escalation trial showed that CD19-targeting CAR T-cells were feasible and safe in R/R B-ALL [24]. At Memorial Sloan Kettering Cancer Center (MSKCC), a CD19-28z CAR T-cell was evaluated in a phase 1 trial of 53 adults with R/R B-ALL. The CR rate was 83%, and the minimal residual disease (MRD)-negative CR rate was 67%. At a median follow-up of 29 months, the median EFS was 6.1 months, and the median OS was 12.9 months. The authors observed that patients with low burden of disease (less than 5% bone marrow blasts) before CAR T-cell infusion had higher remission duration and survival with a median EFS of 10.6 months and a median OS of 20.1 months [25].

### 2.1. Tisagenlecleucel

Tisagenlecleucel (CTL019) is a second-generation, autologous anti-CD19 CAR T-cells medication constructed with activating CD3ζ and costimulatory signals (4-1BB). In a phase 1–2 trial of the Children’s Hospital of Philadelphia, tisagenlecleucel was studied in 30 children and adult patients and was associated with high CR rates (90%), and durable responses up to 24 months were reported [28]. The ELIANA trial, a phase 2 trial, evaluated CTL019 in pediatric and young adults with B-cell ALL in 25 sites and included 75 patients. The overall remission rate within 3 months was 81%, with negative MRD assessed by flow cytometry. The 6-month and 12-month EFS rates were 73% and 50%, respectively, and the 6-month and 12-month OS rates were 90% and 76%. Cytokine release syndrome (CRS) occurred in 77% of patients, while neurologic events were reported in 40% of patients [19]. Based on these results, the FDA approved tisagenlecleucel for the treatment of patients up to 25 years of age with B-ALL that is refractory or in second or later relapse in August 2017. The 3-year update showed that the median EFS was 24 months and that the median OS was not reached. The 3-year EFS and OS rates were 44% and 63%, respectively. There was no new of unexpected long-term adverse events confirming the favorable long-term safety of tisagenlecleucel [33].

### 2.2. Brexucabtagene Autoleucel (KTE-X19)

Brexucabtagene autoleucel (KTE-X19) is an autologous CD19-targeting CAR T-cell therapy that was initially approved for the treatment of patients with R/R mantle cell lymphoma. ZUMA-3 is a phase 1–2 trial evaluating KTE-X19 in adult patients with R/R B-ALL. The recommended phase 2 dose was 1 × 10^6^ cells per kg, and the overall CR or CR with incomplete hematological recovery (CRi) was 83% in the phase 1 trial [34]. These results were confirmed in the phase 2 cohort with a CR/CRi rate of 71% (39/55 patients) at a median follow-up of 16.4 months. The median duration of remission, RFS, and OS were 12.8 months, 11.6 months, and 18.2 months, respectively. CRS of grade ≥ 3 was observed in 24% of patients (13/55), while neurological events of grade ≥ 3 occurred in 25% of patients (14/55) [20]. Based on these results, the FDA approved KTE-X19 for adult patients with relapsed and/or refractory B-cell ALL.

## 3. Real-Life Experience

Available data regarding real-life experience of CD19-targeting CAR T-cells are summarized in Table 2. In a real-life experience from the Pediatric Real World CAR Consortium (PRWCC), outcomes with tisagenlecleucel were similar to the phase 2 pivotal trial. The infused cohort had an 85% CR rate and 12-month OS, and EFS rates were 72% and 50%, respectively, at a median follow-up of 335 days. Grade 3 or higher CRS and neurotoxicity occurred in 21% and 7% of patients, respectively. Moreover, the authors showed that high burden of disease, defined as central nervous system (CNS) or extramedullary disease (EMD) or 5% or higher bone marrow lymphoblasts, was associated with inferior outcomes compared with patients with lower burden of disease or no detectable disease [35]. Furthermore, Pasquini et al. reported the largest retrospective cohort of pediatric patients with B-ALL or non-Hodgkin lymphoma (NHL) treated with tisagenlecleucel. Overall, 255 patients had B-ALL, and the initial CR rate was 85.5%, which is comparable to the ELIANA results. The 12-month EFS and OS rates were 52% and 77% in this population. Grade 3 or higher CRS and neurotoxicity occurred in 16% and 9% of patients. It is noteworthy that, in this cohort, 6% of treated patients were younger than 3 years, while children of less than 3 years were excluded from the ELIANA trial [36]. More recently, a large real-world experience of CD19-targeting CAR T-cells from the Acute Leukemia Working Party of the European Society for Blood and Marrow Transplantation (EBMT) was reported. A total of 118 adult patients with B-ALL were infused, and the median age was 23.8 years. At a median follow-up of 12.4 months, the CR rate was 91%. The majority of patients (82%) were not in CR when CAR T-cells were infused. The 1-year OS rate was 88.9% in patients who were in CR when they received CAR T-cells and 61.9% for those who were not in CR. The 12-month LFS was 65.8% in patients with CR and 38.7% in those who were not in CR [37]. Moreover, a recent meta-analysis of published and unpublished clinical trials concerning adult or pediatric patients with R/R B-ALL and treated with anti-CD19 CAR T-cell therapy between 1 January 2012 and 14 April 2020 was reported. Studies published in languages other than English, studies with insufficient data, and studies with two patients or fewer were excluded. Overall, 953 patients were included in the final analysis. The pooled CR rate was 80% with a similar activity for children and adults. The 12-month progression-free survival (PFS) rate was 37%. There was no statistical difference in terms of pooled CR rate according to CAR T-cell construct type or single-chain variable fragment clone. However, patients who received autologous CAR T-cells had improved CR in comparison with those treated with allogeneic CD19-targeting CAR T-cells. The authors did not find any significant difference in the proportion of patients with CRS and neurotoxicity according to anti-CD19 CAR T-cell constructs [38].

## 4. CD19-Targeting CAR T-Cell and Extra-Medullary Disease

Data for CAR T-cell therapy in EMD involvement are limited. Fabrizio and colleagues retrospectively reported the outcome of 184 patients from the PRWCC and treated with tisagenlecleucel. Among them, fifty-five patients had EMD. In patients with CNS involvement, 88% of patients (35/40) achieved CR vs. 66% of patients (10/15) with non-CNS EMD. Patients with CNS involvement (both with and without bone marrow (BM) involvement) had 2-year OS outcomes comparable to those of non-CNS EMD or BM disease only (*p* = 0.41). Furthermore, 12-month RFS was not statistically different between CNS, non-CNS EMD, and BM-only patients (*p* = 0.92). There was no statistically increased toxicity with CNS or non-CNS EMD (*p* = 0.3). The presence of active CNS disease at the time of infusion did not affect patient outcomes [39]. In patients with CNS involvement, a post hoc analysis of pooled data from five clinical trials (Pedi CART19, 13BT022, ENSIGN, ELIANA, and 16CT022) showed that tisagenlecleucel and humanized CART19 (huCART19) are active at clearing CNS disease and are associated with durable responses in children and young adults with R/R B-ALL or lymphocytic lymphoma with CNS involvement. The CR rate on day 28 was similar in the CNS-positive group in comparison with the CNS-negative group (97% versus 94%, *p* = 0.74). There was no significant difference in 2-year RFS (60% vs. 60%, *p* = 0.5) and OS (83% versus 71%, *p* = 0.39). There was also no increasing risk of severe neurotoxicity and CRS [40]. Moreover, in a retrospective analysis of seven patients with R/R B-ALL who received tisagenlecleucel, including six with isolated CNS relapse, the ORR was 100%, and five patients remained in CR at a median of 18 months [41]. Similarly, Jacoby et al. reported in a retrospective analysis the outcome of CD-19-targeted CAR T-cells in a pediatric population with relapsed B-ALL with CNS involvement. Overall, 55 patients were enrolled, of whom 16 had active CNS disease at the time of lymphodepletion. Fifty-one of fifty-four evaluable patients (94%) achieved CR following CAR T-cell therapy. However, 22 patients relapsed following CAR T-cells: 19/43 following 4-1BB-based CARs (12 CNS relapses) and 3/12 after CD28-based CARs with subsequent allogenic HSCT (no CNS relapse). The authors reported that patients treated with tisagenlecleucel for isolated CNS disease had a high incidence of a subsequent CNS relapse (six of eight patients). CRS and neurotoxicity were reported in 65% and 38% of patients, respectively [42].

These findings were confirmed in a recent analysis of 48 patients with R/R B-ALL with CNS involvement and treated with CD19-targeting CAR T-cells. The ORR was 87.5% in BM disease, and the remission rate of the CNS disease was 85.4%. At a median follow-up of 11.5 months, the median EFS was 8.7 months, and the median OS was 16.0 months. The 12-month cumulative incidence of relapse (CIR) was 31.1% and 11.3% in BM and CNS diseases, respectively (*p* = 0.040). Grade 3 or higher CRS and neurotoxicity occurred in 18.8% (9/48) and 22.9% (11/48) of patients [43].

Taken together, these studies suggest that CAR T-cell therapy represents a promising strategy for patients with R/R B-ALL with CNS relapse associated or not with bone marrow disease.

## 5. Toxicity of CAR T-Cells

The two major reported toxicities of CAR T-cell therapy are CRS and ICANS, both of which are due to the mechanism of action of CD19-targeting CAR T-cell therapy, and both are potentially life-threatening. Concerning CRS, it is reported in the majority of patients with ALL and ranging from 77% to 93% [19,21]. The more common clinical features are fever, hemodynamic instability, and hypoxia, and its severity is graded according to the American Society for Transplantation and Cellular Therapy consensus grading scale. The main differential diagnosis is neutropenic fever, and broad-spectrum intravenous antibiotics should be introduced [44]. In comparison with other B-cell malignancies, the incidence of CRS is high across all trials of CD19-targeting CAR T-cell therapy [45]. In patients with B-ALL, the burden of disease is a strong predictor for CRS [20,25,28,46,47]. The management of CRS depends on its grade and severity. It can be self-limited, requiring antipyretics and intravenous fluids, or serious, requiring corticosteroids or tocilizumab (an interleukin 6 receptor antibody). The use of pre-emptive tocilizumab has also evaluated been to prevent CRS. In one study reported by the Children’s Hospital of Philadelphia, patients with high burden of disease were given tocilizumab for persistent fever. Tocilizumab showed a reduction in grade 4 CRS from 50% to 27% [48]. Earlier, tocilizumab was also evaluated in Seattle Children’s Center, which resulted in a lower incidence of severe CRS, and there was no impact on response [21]. ICANS occurs in 20% to 60% of patients treated with CD19-targeting CAR T-cell therapy typically 3 to 5 days after infusion [49]. The pathophysiology of ICANS is not well known, but some factors have been incriminated such as inflammatory cytokines that increase vascular permeability, increased cytokines in the cerebrospinal fluid, and endothelial activation that leads to blood–brain barrier damage [50]. High burden of disease, CD28-CAR T-cell products, higher doses of CAR T-cells, early and severe CRS, and low platelet count are important risk factors for grade 3 or higher ICANS [51]. The current management of ICANS is based on the use of corticosteroids. The role of tocilizumab for the management of ICANS is not clear, and some data suggest that it may lead to ICANS through an increase in circulating IL-6 [52]. Intensive care unit transfer should be considered for grade 2 or higher ICANS. More recently, other toxicities associated with CAR T-cells such as prolonged hematological toxicity (PHT), B-cell aplasia, and late infections have been reported. Prolonged hematological toxicity (PHT), commonly defined as the presence of severe anemia, neutropenia, or thrombopenia on day 28 or 30 post infusion of CAR T-cells, is a new concern in patients with R/R ALL treated with CAR T-cells. In a pooled analysis from the ELIANA and ENSIGN trials, the median time to resolution of grade 3/4 neutropenia was 2.0 months, thrombocytopenia was 1.9 months, and 1.0 months for anemia [53]. In the ZUMA-3 trial, prolonged grade 3/4 neutropenia, thrombocytopenia, and anemia occurred in 25%, 18%, and 7% of patients, respectively [20]. All patients achieving CR/CRi presented B-cell aplasia and managed with immunoglobulin replacement therapy per institutional guidelines [53]. Both hypogammaglobulinemia and prolonged cytopenias can contribute to increased risk of late infections in patients treated with CAR T-cells. For example, in the ELIANA trial, 18 out of 40 patients who presented prolonged neutropenia beyond day 28 developed grade 3 or 4 infection with notably 18% of late deaths (more than 30 days following infusion) that were infection-related [19]. Furthermore, in the pooled analysis previously mentioned, 14% of patients (18/126) presented infections 4 to 8 weeks post infusion, including 8% of grade 3/4, and 12% of patients with more than 1 year of follow-up reported grade 3/4 infections more than 1 year post infusion [53].

## 6. Mechanisms of Relapse following Anti-CD19 CAR T-Cells

Relapse of B-ALL following anti-CD19 CAR T-cell therapy can be globally divided into two groups using the flow cytometry evaluation of the expression of CD19: CD19-positive (70–80% of patients) and CD19-negative relapses (20–30% of patients) [22]. In a recent report, Schultz and colleagues reported that CD19-negative relapse was associated with significantly lower OS in comparison with those who experienced CD19-positive relapse (12-month OS rate 30% vs. 68%, respectively, *p* = 0.0068) [54].

### 6.1. CD19-Positive Relapse

CD19-positive relapses are in general due to low potency and limited in vivo persistence of the infused CAR T-cells. Multiple factors have been reported to limit the potency and the efficacy of CAR T-cells such as poor long-term persistence, immune tumor microenvironment, and intrinsic dysfunction associated with T-cell exhaustion. The role of the costimulatory domain in CD19-positive relapse has been investigated. In fact, several reports about CD19-targeting CAR T-cells with a 4-1 BB costimulatory domain have demonstrated a strong correlation between CAR persistence (indirectly related to B-cell aplasia) and CD19-positive relapses [19,21,27,30,55]. In an analysis of efficacy of tisagenlecleucel in patients with R/R B-ALL from two pediatric trials (ELIANA and ENSIGN), the authors showed that patients who experienced CD19-positive relapses had more rapid loss of tisagenlecleucel persistence in comparison with those who achieved durable remissions [56]. In another pediatric trial form Seattle Children’s Research Institute evaluating another 4-1 BB CAR T-cell, the authors showed that the longer duration of B-cell aplasia was significantly correlated with the durability of remission [21]. Another trial demonstrated that patients presenting CD19-positive relapse had shorter median persistence of 4-1 BB anti-CD19 CAR T-cells compared with those with CD19-negative relapse (2.5 months versus 6 months, respectively) [57]. However, the correlation between outcome and persistence of CAR T-cells using the CD28 costimulatory domain is less clear, and the available data showed that CD28 CAR T-cells had limited persistence [20,25,26]. In the ZUMA-3 study, 79% of patients had no CAR T-cells detectable with evaluable samples by 6 months, and B-cell recovery was observed in all patients with ongoing response at 12 months [20]. Park and colleagues from MSKCC reported that the median persistence of CD28 anti-CD19 CAR T-cells was 14 days (range, 7–138 days) and that the duration of persistence did not correlate with survival [25]. However, data from recent meta-analysis were contradictory. In a recent systematic review and meta-analysis of Anagnostou and colleagues, 35 studies met the eligibility criteria, and 953 patients were included in the analysis. The pooled CR was 80%, and this CR rate did not significantly differ according the CD19-targeting CAR T-cell construct type or single-chain variable fragment [39]. Another hypothesis to explain disease relapse is the immune-mediated rejection of CAR T-cells containing murine domain. Consequently, humanized anti-CD19 scFv domains were used in the development of CAR T-cells that may bypass this rejection. In a recent report, huCART19 was evaluated in children and young adults with R/R B-ALL. Overall, 74 patients were treated in two cohorts: with (retreatment, n = 33) or without (CAR-naive, n = 41) prior CAR exposure. Infusion with huCART19 was associated with durable responses with long-term persistence of CAR, including in patients who failed prior CAR T-cell therapy. The overall response rate at 1 month after infusion of CAR T-cells was 98% in the treatment-naïve group and 64% in the retreatment cohort. The RFS rates at 12 months were 84% in CAR-naive and 74% in retreatment cohorts. The authors also showed that earlier B-cell recovery was associated with worse RFS and that the cumulative incidence of B-cell recovery by 6 months was lower with huCART19 in comparison with historical groups of tisagenlecleucel patients (15 vs. 29%), and it was not statistically significant [55].

### 6.2. CD19-Negative Relapse

Concerning CD19-negative relapse, it occurs when the CD19 antigen is lost through mutation or epigenetic alterations especially in pre-existing leukemia subclones [58,59,60]. Immune pression selection has been described as a CD19-negative relapse mechanism. It has been demonstrated that CD19-negative leukemic cells were present before the infusion of CAR T-cells in patients with CD19-negative B-ALL relapse after CAR T-cell therapy using single-cell RNA sequencing [59]. Moreover, Bueno et al. found that CD34 + CD19-CD22+ leukemic cells were already present at diagnosis and relapse in the bone marrow samples of 70% of patients with B-ALL and that this frequency doubles in patients who achieved CR after CD19-targeting CAR T-cells [61]. Alternative splicing and acquired mutations are another mechanism of CD19-negative relapse. Orlando and colleagues found mutations in the CD19 domain of 12 patients with CD19-negative relapse after treatment with CAR T-cells [23]. An interesting approach to limit CD19-negative relapse following CD19 CAR T-cells is the infusion of CAR T-cells that target other antigens such as CD20 or CD22, whether using bispecific CAR T-cell targeting more than one antigen or infusing two different products with distinct targets (discussed later).

### 6.3. Role of Previous Blinatumomab

Another concern regarding the efficacy of CD19-targeting CAR T-cells is prior treatment with blinatumomab, a bispecific engaging T-cell targeting CD3 and CD19. It has been thought that blinatumomab could adversely affect the efficacy of CD19-targeting CAR T-cells due to a similar mechanism of action and targeting. For example, in the ELIANA trial, patients treated with blinatumomab or other CD19-targeted therapies were excluded from the trial. However, the use of blinatumomab for the treatment of B-ALL significantly increased, and a higher proportion of patients treated with blinatumomab was included in recent trials. In the ZUMA-3 trial, nearly 45% of the adult patients who received KTE-X19 had prior treatment with blinatumomab [20]. In a retrospective analysis of the outcomes of 166 patients treated with CD19-targeting CAR T-cells at the Children’s Hospital of Philadelphia, the CR rate was 93%, and 67 patients experienced disease recurrence, including 39 patients with CD19-negative leukemia. In this report, prior treatment with blinatumomab was significantly associated with a higher rate of failure to achieve MRD-negative disease and a higher risk for CD19-negative relapse [62]. However, Myers and colleagues showed in a retrospective analysis that blinatumomab-naïve patients and blinatumomab responders had comparable CR rates (93.5% and 92.9%, respectively) and that blinatumomab nonresponse was independently associated with worse RFS and EFS [63].

## 7. Overcoming Resistance to CD19-Targeting CAR T-Cells

### 7.1. CD22 CAR T-Cell Therapy

Fry et al. reported the results of the first clinical experience of CD22-targeting CAR T-cells in a phase I trial of children and adults with R/R B-ALL. Overall, 55 patients with B-ALL were treated with CD22-targeting CAR T-cells, of whom 51 previously failed CD19-targeted therapy. The CR rate was 70%, and the median OS was 13.4 months (95% CI, 7.7–20.3 months). The safety profile was comparable to CD19-targeting CAR T-cells [64,65]. Pan and colleagues published the results of anti-CD22 CAR T-cells in 34 pediatric and adult patients with R/R B-ALL who failed previous CD19-targeting CAR T-cell therapies. Overall, 24 of 30 (80%) evaluable patients on day 30 achieved CR or Cri, accounting for 70.5% of the intention-to-treat population. Among patients in CR, seven received no further treatment, while 11 patients underwent ASCT. The 12-month LFS rate in transplanted patients was 72%. It has been demonstrated that CD22 antigen loss or mutation was not associated with relapsed disease [66]. More recently, Jeyakumar and colleagues reported the results of a phase 1b trial of CD22-targeting CAR T-cells at Stanford University. Sixteen heavily pretreated patients with a median of five prior lines of treatment were infused. Thirteen patients previously received anti-CD19 therapeutic agents. Twelve patients (75%) achieved CR or CRi, and nine patients (56%) had negative MRD using flow cytometry. Eleven patients (72%) presented CRS with only one patient with grade 3 CRS, and ICANS occurred in one patient (6%) [67]. Furthermore, Myers et al. presented the results of a phase 1 trial evaluating novel CD22/4-1 BB CAR T-cells using a short scFv linker and called CART22-65s in patients with relapsed CD19-negative B-ALL after failure of CD19-targeted CAR T-cells (NCT02650414). Nineteen patients were enrolled, and seventeen patients were infused. The median dose of CAR T-cells infused was 4 × 10^6^ CART22-65s cells per kilogram of body weight. Thirteen patients (77%) achieved CR on day 28, of whom ten patients presented as MRD-negative by flow cytometry. Overall, five patients underwent consolidative HSCT. At a median follow-up of 29 months, median RFS, EFS, and OS were 5.3 months, 5.8 months, and 16.5 months, respectively. CRS occurred in 15 patients (88%), all of grade 1–2, and ICANS occurred in 6 patients (35%) [68]. Table 3 summarizes the major trials of CD22-targeting CAR T-cells.

### 7.2. Bispecific CD19 and CD22 CAR T-Cells

Bispecific CAR T-cells that target CD19 and CD22 antigens may overcome the risk of CD19-negative relapse. In a phase 1 trial of six patients treated with bispecific CAR T-cell therapy targeting CD19 and CD22 at a dose ranging from 1.7 to 3.0 × 10^6^ CAR T-cell per kilogram of body weight, the MRD-negative CR rate was 100% (6/6). Two patients relapsed at 3 and 10 months, respectively. CRS occurred in 100% of patients, but no grade 3 or higher CRS was reported. No neurotoxicity was reported [69]. In another phase 1 trial (CAR-T GC022), 17 patients with R/R B-ALL, including 4 who failed CD19-targeting CAR T-cell therapy, were enrolled. In patients who received a low dose (2.5–5 × 10^5^/kg), the CR rate was 25% (1/4) with MRD-positive disease, and the CR rate was 100% in the seven patients who received a medium dose (1.25 × 10^6^/kg). All patients presented CRS, and no ICANS has been reported [70].

Spiegel and colleagues reported that bispecific tandem CAR targeting CD19 and/or CD22 in a phase I trial was associated with a 100% response in 17 adult patients with B-ALL including 88% MRD-negative complete remission (CR) 10^−4^ bone marrow sensitivity. Fifty percent of patients (5 out of 10) with B-ALL presented a CD19^-/lo^ relapse. At a median follow-up of 9.3 months, the median OS and PFS were 11.8 months and 5.8 months, respectively [71].

The AMELIA trial is another phase 1 trial evaluating AUTO3, bispecific autologous CAR T-cells targeting CD19 and CD22. Overall, 15 pediatric and young adult patients with B-ALL were enrolled, and 14 of them were CAR-T-cells-naive. AUTO3 was associated with a good safety profile, and no dose-limiting toxicities were reported. CRS occurred in 80% of patients (12/15) with no grade 3 or higher CRS, and neurotoxicity was reported in four patients (27%), grade 1 in all. At 1 month, the CR/CRi rate was 86% (13/15). The 12-month OS and EFS rates were 60% and 32%, respectively. The authors concluded that relapses were probably due to the limited long-term persistence of AUTO3 [72]. Schultz and colleagues presented at the 2022 American Society of Hematology annual meeting a long-term follow-up of a single-institution phase I trial evaluating CD19/22 bispecific CAR T-cells. The CAR incorporated the FMC63 CD19 and M971 CD22 single-chain variable fragments (scFvs) and 4-1BB costimulatory and CD3-zeta endodomains. Overall, 17 pediatric and young adult patients were enrolled, and 15 patients received CAR T-cells. The median age was 13 years. The CR rate was 93% (14/15), and the negative MRD by flow cytometry was obtained in 87% of patients (13/15). Interestingly, 12 patients (80%) received post CAR T-cell HSCT, 3 following relapse and 9 while in CR. The 1-year EFS rate was 64%. The 1-year, 2-year, and 3-year OS rates were 93%, 83%, and 71%, respectively. The authors concluded that bispecific CAR T-cells are effective with a CR rate of 93% and favorable long-term OS and EFS when coupled with HSCT (discussed later) [73]. Recently, the results of a phase 1 trial concerning novel bicistronic CD19/22 CAR T-cells were reported. Overall, 20 children and young adults with R/R B-ALL were enrolled. The CR rate of the entire cohort was 60% (12/20) and 71% in patients who were CAR-naïve. The CRS occurred in 50% of patients (10/20), including 15% of grade 3 or higher CRS. ICANS occurred in only one patient (Grade 3). The 12-month RFS in patients who achieved CR was 58% [74].

### 7.3. Sequential Infusion of CAR T-Cells

Sequential infusion of CD19 and CD22 targeting CAR T-cells was also evaluated in patients with R/R B-ALL. Wang and colleagues reported the results of a pilot study of 89 patients with B-cell malignancies and who received a sequential infusion of two single-specific third-generation CD19- and CD22-targeting CAR T-cells. A total of 51 patients with B-ALL were infused, and the MRD-negative response rate was 96%. At a median follow-up of 16.7 months, the median PFS was 13.6 months, and the median OS was 31.0 months [75]. The sequential infusion was also evaluated in a phase 1 trial in a pediatric population with B-ALL. Twenty patients were included, and all of them achieved CR and negative MRD on day 30 after anti-CD19 CAR T-cells and remained in CR with negative MRD before the infusion of CD22-targeting CAR T-cells. No allogenic HSCT was performed. The median LFS and OS were not reached, and the 12-month LFS and OS rates were 79.5% and 92.3%, respectively. Three patients relapsed at 6.6, 6.9, and 11.4 months. Interestingly, CAR T-cells were detected in the cerebrospinal fluid of the patients who were examined [76].

### 7.4. Coadministration of CD19- and CD22-Targeting CAR T-Cell

More recently, the coadministration of CD19- and CD22-targeting CAR T-cells was a novel approach adopted by Wand and colleagues in a phase II trial in patients aged ≤ 20 years with R/R B-ALL. Overall, 194 patients were treated, the CR rate was 99%, and all were negative for MRD. Interestingly, the 12-month EFS rate was 73.5%, irrespective of allogenic HSCT, and the 12-month OS rate was 87.7% at a median follow-up of 11 months. The 12-month EFS in patients who underwent HSCT (78 patients) was 85% versus 69.2% for non-transplant patients (116 patients), which was statistically significant (*p* = 0.03). However, this advantage in EFS was not translated into an OS advantage because of the short median follow-up and the fact that relapsed non-transplant patients were salvageable or still alive with disease. Relapse was reported in 43 patients (22%) and was CD19^+^/CD22^+^ in 24 patients, CD19^−^/CD22^+^ in 16 patients, and CD19^−^/CD22^−^ in only one patient [77].

### 7.5. Allogenic CAR T-Cell Therapy

Another important achievement in the evolution of CAR T-cells is the development of allogenic universal CAR T-cells (UCAR T) produced from healthy donors and manipulated using transcription activator-like effector nucleases (TALENs). Preliminary results of UCART-T19, an allogenic CD19-targeting 41BB CAR T-cell, from two studies (NCT02808442 and NCT02746952) were recently reported. The lymphodepletion consisted of fludarabine and cyclophosphamide with or without alemtuzumab (a CD52-targeting monoclonal antibody). Overall, 21 patients (7 children and 14 adults) were enrolled, and 67% (14/21) achieved CR or CRi on day 28 after infusion, of whom 10 patients underwent allogenic HSCT. The median duration of response was 4.1 months, and the 6-month PFS and OS rates were 27% and 55%, respectively. CRS and ICANS of any grade occurred in 91% and 38% of patients, respectively. Two patients presented grade 1 cutaneous graft vs. host disease (GVHD) [78]. The BALLI-01 (NCT04150497) is a phase 1 trial evaluating UCAR-T22, allogenic CD22-targeting 41BB CAR T-cells, in patients with R/R B-ALL. Eleven patients received UCAR-T22 infusion. The median age was 30 years (range 20–61 years). Only two patients (16%) presented anti-leukemic activity at day 28. Three patients presented CRS, no ICANS was reported, and one patient presented grade 2 cutaneous GVHD. Overall, UCAR-T22 was associate with limited activity and a tolerable safety profile [79].

### 7.6. Role of Bridging Therapy and Lymphodepletion Regimens

The role of bridging therapy on the efficacy of CD19-targeting CAR T-cells is not well recognized. Shahid et al. found that patients who received two or more cycles of bridging therapy had significantly lower OS and higher grade ≥ 3 infection than those who received only one cycle in a small group of children and young adults with R/R B-ALL [80]. Moreover, data from MSKCC showed that disease status at the 3-month landmark was significantly associated with OS and that both patients who presented a low burden of disease and patients who responded to bridging chemotherapy had favorable outcomes in comparison with patients with persistent morphologic disease [81]. These results were contradictory with those reported in patients with lymphoma. Johnson et al. found that bridging therapy use is not associated with differences in ORR, CR rate, or PFS but is associated with worse OS [82]. Recently, Roddie and colleagues reported that patients with lymphoma who achieved complete or partial response to bridging therapy had a 42% reduction in the risk of progression or death compared to nonresponding patients [83].

The role of lymphodepletion regimens had also been evaluated in patients treated with CD19-targetong CAR T-cells. In fact, the addition of fludarabine to cyclophosphamide has improved the kinetics of CAR T-cells as well as the initial response [21]. Turtle and colleagues showed that nearly 90% of patients who received fludarabine-containing regimens achieved CR with no evidence of relapse, while 58% of patients without fludarabine presented disease relapse [48]. Moreover, in children and young adults with B-ALL, patients with higher cumulative fludarabine exposure during lymphodepletion had an 11-month improvement in LFS compared with patients in the lower group, and the 1-year CD19+ recurrence rate decreased from 100% to 27% [84].

### 7.7. Consolidative Allogenic Stem Cell Transplantation

The role of consolidative allogenic HSCT following CAR T-cells is controversial. In fact, two critical questions arise regarding consolidative HSCT. The first concern is the benefit of HSCT in fit patients who achieve MRD-negative CR following CD19-targeting CAR T-cells given allogenic HSCT toxicity. The other issue concerns CAR T-cells with prolonged functional persistence that would be eliminated with HSCT, leading to the loss of their anti-leukemic effect. Unfortunately, to date, there are no clinical trials that randomized patients to consolidative allogenic HSCT versus observation following CAR T-cell therapy.

The NCI reported the results of a phase 1 trial of children and young adults with B-ALL evaluating autologous CD19-targeting CAR T-cells. In this study, 21 of 28 patients (75%) with MRD-negative CR underwent allogenic HSCT. Interestingly, the median OS of these patients was 70.2 months compared with 10.5 months for the entire cohort (50 patients). For the two patients who proceeded to allogenic HSCT relapse, the CIR after HSCT was 9.5% at 24 months, and the 5-year EFS was 61.9%. Seven patients with MRD-negative CR that did not undergo consolidative HSCT relapsed at a median of 152 days post infusion of CAR T-cells. [26]. However, the data reported from MSKCC showed that there was no significant difference in OS in patients who did or did not undergo consolidative HSCT following CAR T-cells [25]. Haploidentical HSCT following CAR T-cells was recently evaluated in children and young adults with R/R B-ALL. Fifty-two patients underwent haploidentical HSCT after achieving CR following CAR T-cell therapy. At a median follow-up of 24.6 months, the 12-month EFS, OS, and CIR rates were 80.1%, 92.3%, and 14.1%, respectively. The median time from CAR T-cells to haplo-HSCT was 61 days. The authors found that HSCT was not associated with increased risk of 24-month cumulative incidence of GVHD, treatment-related mortality, or infection. Moreover, pre-transplant MRD-positive disease was an independent factor associated with poor OS [85].

Zhao and colleagues reported a multicenter retrospective study of haploidentical HSCT for R/R B-ALL. A total of 122 patients received CD19-targeting CAR T-cells, including 55 patients who underwent haploidentical HSCT and 67 patients without subsequent transplantation. Patients who underwent HSCT had a significantly higher 2-year OS rate and LFS when compared with the non-transplant group (77% vs. 36.4%, *p* < 0.001, and 65.6% vs. 32.8%, *p* < 0.001, respectively). The authors found that MRD-positive disease at transplantation is an independent factor associated with poor LFS, OS, and high CIR rates [86]. Summers and colleagues evaluated the impact of consolidative allogenic HSCT among pediatric patients and young adults enrolled in the phase 1/2 (PLAT-02) trial. A total of 50 patients with MRD-negative CR were evaluable, of whom 23 patients underwent consolidative allogenic HSCT. The authors found that LFS was superior in patients who underwent HSCT compared to those who did not (HR, 0.31; *p* = 0.01), but no significant difference in OS rates was observed (HR: 0.62, *p* = 0.38). Interestingly, the relapse risk following a short duration of B-cell aplasia is mitigated by consolidative HSCT [87]. In a Chinese prospective trial, 47 patients with R/R B-ALL received CD19-targeting CAR T-cells, and all patients achieved MRD negativity. Twenty-one patients underwent consolidative allogenic HSCT at a median 44 days after CAR T-cells. Overall, patients with allogenic HSCT had significantly prolonged EFS and RFS (*p* < 0.05), but these results were not translated into OS advantage between the two groups [58].

## 8. Conclusions

CD19-targeting CAR T-cells changed the treatment paradigm of B-ALL in pediatric and adult patients with an acceptable and manageable safety profile. However, nearly 50% of patients relapsed in the year following CAR T-cell perfusion. Disease relapse can be CD19-positive and CD19-negative. Several efforts have been made to improve the clinical outcomes of CAR T-cells such as the use of bispecific and/or sequential infusion of CD19- and CD22-targeting CAR T-cells. The role of consolidative allogenic HSCT is not well established yet. Table 4 summarizes the major ongoing clinical trials of CAR T-cells for the management of patients with B-ALL.

## Figures and Tables

**Table 1:** Major prospective trials of CD19-targeting CAR T-cells.

Reference	Costimulatory Domain	Study Population and Median Age	Design	N	CR (%) MRD-CR (%)	Survival	CRS (%) Grade ≥ 3 (%)	ICANS (%) Grade ≥ 3 (%)
NCT01593693 [24,26]	CD28	Pediatric and young adults 13.5 y	Phase 1	50	62% 56%	Median OS: 10.5 m	70% 22%	20% 8%
NCT01865617 [27]	4-1 BB	Adults 39 y	Phase 1–2	53	85% 85%	NA	75% 19%	23% 23%
NCT01044069 [25]	CD28	Adults 42 y	Phase 1	53	83% 67%	Median OS: 12.9 m Median EFS: 6.1 m	85% 26%	44% 42%
NCT01626495 and NCT01029366 [28]	4-1BB	Pediatric and adult 14 y	Phase 1–2	30	90% 73%	6-month OS: 78% 6-month EFS: 67%	100% 27%	43%
NCT02435849 (ELIANA) [19]	4-1 BB	Pediatric and young adult 11 y	Phase 2	75	81% 81%	12-month OS: 76% 12-month EFS: 50%	77% 46%	40% 13%
NCT02614066 (ZUMA-3) [20]	CD28	Adult patients 40 y	Phase 2	55	71% 71%	Median OS: 18.2 m Median RFS: 11.6 m	89% 24%	60% 25%
NCT02028455 (PLAT-02) [21]	4-1 BB	Pediatric and young adult 12.3 y	Phase 1–2	43	93%	12-month OS: 69.5% 12-month EFS: 50.8%	93% 23%	49% 21%
NCT01860937 [29]	CD28	Pediatric and young adult 13.5 y	Phase 1	25	75%	NA	80% 16%	72% 28%
NCT02030847 and NCT01029366 [30]	4-1 BB	Adults 33.8 y	Phase 1–2	35	69%	Median OS: 19.1 m Median EFS: 5.6 m	94% 72%	43% 6%
NCT02735291 [31]	4-1 BB	Pediatric and adults 22 y	Phase 2	47	81% 79%	12-month OS: 53% 12-month RFS: 45%	83% 23.4%	4.3% 2.1%
NCT02935257 (ALLCAR19) [32]	4-1 BB	Adults 41.5 y	Phase 1	20	85%	12-month OS: 64% 12-month EFS: 48%	55% 0%	20% 15%

N: number; CR: complete remission; MRD-: minimal residual disease negative; CRS: cytokine release syndrome; ICANS: immune effector cell-associated neurologic syndrome; OS: overall survival; EFS: event-free survival; RFS: relapse-free survival; NA: not available.

**Table 2:** Real-life experience of CD19-targeting CAR T-cells.

Reference	Drug	Study Population and Median Age	N	CR (%) MRD-CR (%)	Survival	CRS (%) Grade ≥ 3 (%)	ICANS (%) Grade ≥ 3 (%)
PRWCC [35]	Tisagenlecleucel	Pediatric and young adults 12 y	185	85% 80%	12-month OS: 72% 12-month EFS: 50%	63% 21%	21% 7%
Pasquini et al. [36]	Tisagenlecleucel	Pediatric 13.2 y	255	85.5% 99.1% (115/116)	12-month OS: 60.9% 12-month EFS: 52.4%	55% 16.1%	27% 9%
Brissot et al. [37]	CD19-targeting CAR T-cells	Adults 23.8 y	118	91% NA	12-month OS: 88.9% in CR 12-month OS: 61.9% in non-CR	88% NA	NA NA
Anagnostou et al. [38]	CD19-targeting CAR T-cells	Pediatric and adults NA	953	80% 72%	12-month OS: 58%	82% 26%	29% 12%

N: number; CR: complete remission; MRD-: minimal residual disease negative; CRS: cytokine release syndrome; ICANS: immune effector cell-associated neurologic syndrome; OS: overall survival; EFS: event-free survival; NA: not available.

**Table 3:** Major trials with CD22-targeting CAR T-cells.

Reference	Costimulatory Domain	Study Population and Median Age	Design	N	Prior CD19 CAR	CR (%) MRD-CR (%)	Survival	CRS (%) Grade ≥ 3 (%)	ICANS (%) Grade ≥ 3 (%)
NCT02315612 [65,66]	4-1 BB	Pediatric and young adults 17.5 y	Phase 1	58	62%	70% 43%	Median OS: 13.4 m	86% 76%	33% 2%
ChiCTR-OIC-17013523 [67]	4-1 BB	Pediatric and adults 10 y	NA	34	91%	71% 53%	NA	91% 3%	18% 0%
NCT04088890 [68]	NA	Pediatric and adults 23 y	Phase 1b	16	58%	75% 56%	NA	72% 6%	6% 6%
NCT02650414 [69]	4-1 BB	Pediatric and young adults 16 y	Phase 1	17	94%	77% 59%	Median OS: 16.5 m Median EFS: 5.8 m	82% 0%	35% 0%

**Table 4:** The major ongoing clinical trials of CAR T-cells for the management of patients with B-ALL.

Trial	Target	Phase	Nb of Patients	Population	Primary Endpoint
NCT03876769	CD19 (tisagenlecleucel)	2	140	First-line high-risk pediatric and young adult with B-ALL with MRD+ at the end of consolidation	DFS
NCT05535855	CD19 (autologous)	1	14	MRD positive at CR1	Frequency of AEs DLTs
NCT04690595	BAFFR (autologous)	1	24	R/R after at least 2 lines including CD19 targeting treatment	Incidence of AEs
NCT04404660 (AUTO1)	CD19 (autologous)	1–2	215	Adults with R/R B-cell ALL	1b: frequency of AEs 2: ORR
NCT02935257 (ALLCAR19)	CD19 (autologous)	1	60	Adults with R/R ALL, DLBCL, CLL, FL, MCL	
NCT05480449 (huCART19 prodigy)	CD19 humanized (autologous)	1–2	89	Pediatric population with R/R disease	Safety ORR
NCT05613348 (CAR19T2)	CD19 humanized	1–2	70	Pediatric ALL	ORR MTD AEs
NCT04609241	CD79b	1	72	Pediatric and adults with R/R ALL or NHL	DLT TEAEs
NCT04781634	CD19/CD22 (autologous)	1–2	40	Pediatric and adult patients with R/R ALL	AEs ORR
NCT04723901	CD19/CD22 (autologous)	1–2	20	Young adult and adult with R/R ALL	CR rate
NCT05225831	CD19/CD22 (autologous)	1	100	Pediatric and adult patients with R/R ALL	AEs CR rate
NCT04049383	CD19/CD20 (autologous)	1	24	Pediatric and young adults with R/R ALL	AEs
NCT04788472	Sequential CD19 and CD22 (autologous)	1–2	50	Young adults and adults with R/R Phi+ ALL	DLT Incidence of TEAEs
NCT04740203	Sequential CD19 and CD22 (autologous)	1–2	50	Young adults and adults with R/R Phi-ALL	DLT Incidence of TEAEs
NCT05164042	CD19 (allogenic)	1–2	20	Young adults and adults with R/R ALL	CR rate
NCT05507827	CD19/CD22 (allogenic)	1	18	Adults with R/R ALL	Safety
NCT05310591 (CAPTiRALL)	Nivolumab + tisagenlecleucel	1–2	26	Pediatric and young adults with R/R ALL after loss of persistence	% of pts with limiting toxicity Efficacy
NCT05418088	CD19/CD20/CD22 (autologous)	1	36	R/R B-cell malignancies including ALL	Recommended dose for phase 2

DLTs: dose-limiting toxicities; AEs: adverse events; CR1: first complete remission; MRD: minimal residual disease; DFS: disease-free survival; R/R: relapsed and/or refractory; B-ALL: B-cell acute lymphoblastic leukemia; CR: complete remission; ORR: objective response rate; NHL: non-Hodgkin lymphoma; DLBCL: diffuse large B-cell lymphoma; CLL: chronic lymphocytic leukemia; FL: follicular lymphoma; MCL: mantle cell lymphoma; MTD: maximum tolerated dose; TEAEs: treatment-emergent adverse events.

## Data Availability

We report most of the data in the tables. Additional details are available on request from the corresponding author.
